# PET in vivo generators ^134^Ce and ^140^Nd on an internalizing monoclonal antibody probe

**DOI:** 10.1038/s41598-022-07147-x

**Published:** 2022-03-09

**Authors:** G. W. Severin, J. Fonslet, L. K. Kristensen, C. H. Nielsen, A. I. Jensen, A. Kjær, A. P. Mazar, K. Johnston, U. Köster

**Affiliations:** 1 The Hevesy Laboratory, DTU Health Technology, Technical University of Denmark (DTU), Frederiksborgvej 399, 4000 Roskilde, Denmark; 2 Department of Clinical Physiology, Nuclear Medicine & PET and Cluster for Molecular Imaging, Rigshospitalet and University of Copenhagen, Copenhagen, Denmark; 3 Minerva Imaging, Copenhagen, Denmark; 4 Monopar Therapeutics, Inc., Wilmette, IL USA; 5 ISOLDE, CERN, Geneva, Switzerland; 6 Institut Laue-Langevin, Grenoble, France; 7 Department of Chemistry, Michigan State University, East Lansing, MI USA; 8 Facility for Rare Isotope Beams, Michigan State University, East Lansing, MI USA

**Keywords:** Nuclear chemistry, Experimental nuclear physics, Preclinical research

## Abstract

The in vivo-generator radionuclides ^140^Nd (t_1/2_ = 3.4 d) and ^134^Ce (t_1/2_ = 3.2 d) were used to trace a urokinase-type plasminogen activator (uPA)-targeting mouse monoclonal antibody, ATN-291, in U87 MG xenograft tumor-bearing mice. ATN-291 is known to internalize on the uPA/uPA-receptor pair, making it an appropriate targeting vector for investigating the fate of in vivo generator daughters on internalizing probes. *Ante-mortem* and *post-mortem* PET imaging at 120 h post-injection gave no indication of redistribution of the positron emitting daughter nuclides ^134^La and ^140^Pr from tumor tissue (*p* > 0.5). The lack of redistribution indicates that the parent radionuclides ^134^Ce and ^140^Nd could be considered as long-lived PET-diagnostic matches to therapeutic radionuclides like ^177^Lu, ^161^Tb and ^225^Ac when internalizing bioconjugates are employed.

## Introduction

Targeted radionuclide therapy is becoming increasingly successful with long-lived radioisotopes such as ^177^Lu, ^161^Tb, and ^225^Ac. One concern over their use, however, is potential long-term instability of the targeting vector and radiolabeling constructs. Even after uptake and accumulation at disease sites, instability or catabolism may lead to unintended dose re-distribution. For targeted alpha radiotherapy with ^225^Ac, there is an additional concern that arises from the potential for redistribution of radioactive daughters following recoil-induced release of the daughter radionuclide after the initial decay^1^. McDevitt et al. showed that in cases where a targeting molecule bearing ^225^Ac is internalized, the subsequent decays of daughters remain localized, whereas when the vector is not internalized, daughter redistribution causes the targeted therapy to be less potent^2^.

Due to the increasing demand for targeted therapies, it is important to have tools that can accurately predict dosimetry and assess the consequences of chelate-vector instability introduced by chemical interactions or by radioactive decay.

**Figure 1:**
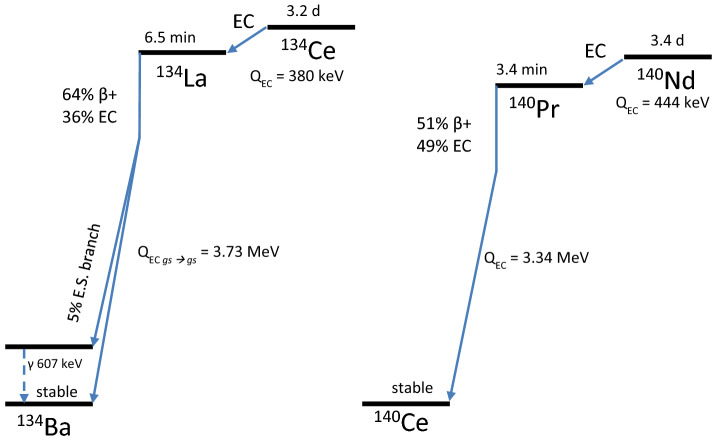
Simplified decay schemes for the ^134^Ce and ^140^Nd decay chains.

The radionuclides cerium-134 (^134^Ce, t_1/2_ = 3.2 d, EC) and neodymium-140 (^140^Nd, t_1/2_ = 3.4 d, EC) provide an interesting opportunity to study the in vivo behavior of long-lived and decay-released radionuclides^3,4^. Both of these long-lived radiolanthanides decay to relatively short-lived positron emitting daughters: lanthanum-134 (t_1/2_ = 6.5 min, 64% β^+^) and praseodymium-140 (^140^Pr, t_1/2_ = 3.4 min, 51% β^+^) respectively (see Fig. 1). Due to the delayed positron emission, ^140^Nd and ^134^Ce, are deemed PET “in vivo generators”^5^. Importantly, for both of these decay chains, the parent EC decay is locally disruptive and results in a separation of the daughter radionuclide from tracer-radioconjugates with roughly 100% efficiency (see, e.g.^6^ and discussion in^5^). In a previous study, we observed that ^140^Nd, when bound to the non-internalizing somatostatin-receptor-targeting molecule, DOTA-LM3, released a freely circulating ^140^Pr ion after EC decay. The circulation of non-ligand-bound ^140^Pr led to PET scans in a preclinical mouse model that were not representative of the DOTA-LM3 biodistribution, but rather showed a convolution of the distribution of the tracer with that of the unbound metal^7^.

The present study is a test-case for performing imaging and biodistribution studies with in vivo generators bound to an internalizing probe, specifically the mouse monoclonal antibody ATN-291. ATN-291, generated by Mazar and coworkers, has promise as a vector for targeted systemic radiotherapy against cells generating and expressing the urokinase-type plasminogen activator, uPA, and its receptor, uPAR. ATN-291 is unique because it targets the uPA-uPAR pair through bound uPA^8^. This implies that a payload-based therapy with ATN-291 could truly target only those cells which are actively breaking down the extracellular matrix ahead of invasion^9^. Importantly, the bound antibody has been shown to internalize during the uPAR recycling process^8,10^. In 2016 Yang and coworkers showed that ATN-291 accumulates preferentially in aggressive xenograft cancer models, where maximum uptake of the antibody is positively correlated with tumor seeding efficiency^11^.

When considering systemic radionuclide therapy as a treatment modality, the process targeted by ATN-291 makes it an ideal vector for delivering short-range, high-LET radiation via alpha, Auger, or low-energy beta emitters to the cells which are actively involved in metastasis formation. If well understood, the radionuclides ^134^Ce and ^140^Nd can act as stand-ins for the therapeutic lanthanides and actinides in PET-based dosimetry determinations and theranostics, especially when the chosen therapeutic isotope has a long half-life. Notably, ^134^Ce is recognized as an imaging surrogate for ^225^Ac due to similar valence configurations and ionic radius of the 3+ ions, and this similarity has motivated extensive research into the applicability of using ^134^Ce and ^225^Ac as a matched pair^4^. In the absence of ^134^Ce and ^140^Nd, PET-based matched-pair imaging is not possible at late time points due to the short half-lives of positron emitting lanthanides^12^. ^134^Ce and ^140^Nd have the added advantage that, in certain circumstances, they can also provide information about the redistribution of daughter radionuclides following decay, or alternatively the short-term fate of unstably bound lanthanides.

The data presented in this work are consistent with the hypothesis that the lanthanide daughters, ^134^La and ^140^Pr, of the in vivo generators ^134^Ce and ^140^Nd, when internalized, have a nearly equivalent distribution to the vector-bound parents (rather than redistributing, as was observed with the non-internalizing probe). These results show promise for short-range targeted radiotherapies using ATN-291 (or other internalizing mAbs) as the carrier molecule. Additionally, ^134^Ce/^134^La and ^140^Nd/^140^Pr are promising as theranostic matched pair PET radiolabels for therapies with long-lived lanthanides and actinides like ^169^Er, ^161^Tb, ^177^Lu, and ^225^Ac.

## Materials and methods

### Isotope production

^134^Ce and ^140^Nd were produced at ISOLDE-CERN by 1.4 GeV proton-induced spallation of tantalum foil targets, released on-line by solid state diffusion from the > 2000 °C hot targets, thermally ionized in a tubular tungsten ionizer, accelerated to 30 kV and mass-separated as described previously^7,13–15^. In this case, the A = 150 monoxide sideband was used to collect ^134^Ce, while ^140^Nd was collected at A = 140. Collection was performed by implanting the ions into a thin layer of Zn on a gold foil.

Chemical purification from Zn-coated gold “catcher foils” followed the previously published procedure with minor alterations^7^. Based upon the chemical conditions involved, ^134^Ce was presumed to be in the 3+ oxidation state for the entire procedure, which is consistent with the findings of Bailey et al*.*^4^.

Note: the following procedure was carried out separately and equivalently for the ^134^Ce and ^140^Nd containing foils. The implanted radionuclides were dissolved by etching the Zn with approximately 50–100 μL 0.1 M hydrochloric acid, which was diluted to a total volume of 500 μL with 2 M HCl. Zn(II) was removed from the resulting solution by passing it over 700 mg anion-exchange resin (AG1 × 8, BioRad, chloride form, prepped with an HCl gradient) and washing out the radionuclides with an additional 1.5 mL 2 M HCl. 1 mL ammonium acetate solution (1 M, pH 7) was added to the collected effluent, which was then pH adjusted to 5–6 with 400–500 μL 25% aqueous NH_3_. The pH adjusted solutions were passed over 80 mg hydroxamate functionalized SPE resin (Zr resin, Triskem) which retained the radionuclides. The resins were then washed with 6 mL 100 mM sodium acetate (pH 5.5) and 10 mL of water. Finally, the radionuclides were eluted from the resins with 1.5 mL 0.1 M HCl.

### Antibody conjugation and radiolabeling

The mouse monoclonal antibody ATN-291 (1.5 mg in 290 µL PBS) was reacted with CHX-A”-DTPA-p-Bn-SCN (5 µL, 20 μmol/mL in DMSO, Macrocyclics) after pH adjustment to 9 with aq. sodium carbonate (50 µL, 100 mM). After 1 h of gentle agitation in a 37 °C water bath the solution was passed over a PD-10 gel filtration column (GE Life Sciences) in 100 mM pH 5 sodium acetate, and the large molecule fraction was collected in 1.5 mL of effluent. Efficacy of the conjugation was confirmed through test radiolabeling with ^177^Lu.

100 μL of the ATN-291 solution (protein concentration: 1 μg/μL) was added to a vial containing 500 μL of the radionuclide solutions (0.1 M HCl) and 150 μL 1 M ammonium acetate. The solutions were incubated at 37 °C for 90 min, and again for 60 min after addition of aq. EDTA (100 μL, 50 mM, pH 7) to scavenge unchelated radionuclides, and the radiolabeling efficiency was checked with Al-backed silica TLC plates run in 1:1 acetonitrile and 0.5 M sodium acetate (pH 5). The solutions were then loaded onto a fresh PD-10 column, prepped and eluted with HEPES buffered (10 mM, pH 7.4) isotonic saline. 1.5 mL of eluate, containing the radiolabeled antibodies, were collected for injection.

### Animal model

U87 MG human glioblastoma cancer cells (ATCC HTB-14, LGC Standards) were cultured in EMEM media supplemented with 10% fetal bovine serum and 1% penicillin–streptomycin (Invitrogen) at 37 °C and 5% CO_2_. Cells in their exponential growth phase and at 80–90% confluence were harvested by trypsinization and resuspended in 1:1 EMEM media and Matrigel (BD Biosciences) at 3 × 10^7^ cells/mL. Subcutaneous tumors were established in female NMRI nude mice (Taconic, Denmark) by inoculation of 3 × 10^6^ cells in 100 μL on each flank above the hind limbs in the subcutaneous space. All animals received the same level of care and were distributed amongst the test groups at random, and all inoculations and injections occurred on the same dates. Upon excision, the average tumor mass was 46 ± 17 mg. All animal experiments were performed under a protocol approved by the National Animal Experiments Inspectorate of Denmark. The study was performed in compliance with the ARRIVE 2.0 criteria. A compliance evaluation is included as supplementary information.

### PET-CT scanning

Longitudinal small animal PET/CT imaging (Inveon Multi-modality PET/CT scanner, Siemens) was performed injection of either 2.8–3.1 MBq ^140^Nd-cDTPA-ATN-291 (*n* = 5), 1.8 MBq ^140^Nd-chloride (*n* 2), 1.8–1.9 MBq ^134^Ce-cDTPA-ATN-291 (*n* = *5*) or 1.0 MBq ^134^Ce-chloride (*n* = 2) in 140–150 μL. Mice were anesthetized with sevoflurane (Abbott Laboratories) during injection and PET/CT imaging. PET data was acquired for 900 s at 120 h after injection (as this scan occurs before the animal is sacrificed, it is referred to as the *ante-mortem*, or AM scan). The mice were sacrificed after the AM scan and a 900 s PET acquisition was performed approximately 2 h after sacrifice (this is referred to as the *post-mortem,* or PM scan). Images were reconstructed using a 3D maximum a posteriori algorithm with CT based attenuation correction. CT images were acquired with the following settings: 360 projections, 65 kV, 500 μA, and 400 ms exposure, and reconstructed with an isotropic voxel size of 105 µm. Image analysis was performed using the Inveon Software (Siemens). Regions of interest (ROIs) were drawn manually over tumor regions, heart (blood), kidney, liver and muscle based on the CT images. The uptake of ^140^Nd-cDTPA-ATN-291, ^134^Ce-cDTPA-ATN-291, ^140^Nd-chloride and ^134^Ce-chloride quantified as % injected dose per gram tissue (%ID/g). Conventional ex vivo biodistribution was performed after the PM scan. Tumors and organs were resected, weighted and the radioactivity was counted in a gamma counter (Wizard2, PerkinElmer).

## Results and discussion

### Radiochemistry

0.24 GBq of ^134^Ce and 0.28 GBq ^140^Nd were collected at CERN-ISOLDE and shipped to the Hevesy Laboratory at the Technical University of Denmark. Dissolution and AG1 × 8 and hydroxamate SPE purification the following day yielded 0.17 GBq and 0.19 GBq respectively. Reactions with ATN-291 were monitored by radioTLC, showing that after the initial 90-min incubation, 70% of the ^134^Ce and 85% of the ^140^Nd was bound to the ATN-291. After quenching with EDTA, purification over PD-10 columns indicated a similar radiolabeling yield: 60% of the ^134^Ce and 77% of the ^140^Nd eluted from the PD-10 columns in the large molecule fractions.

### PET scanning and biodistribution

**Figure 2:**
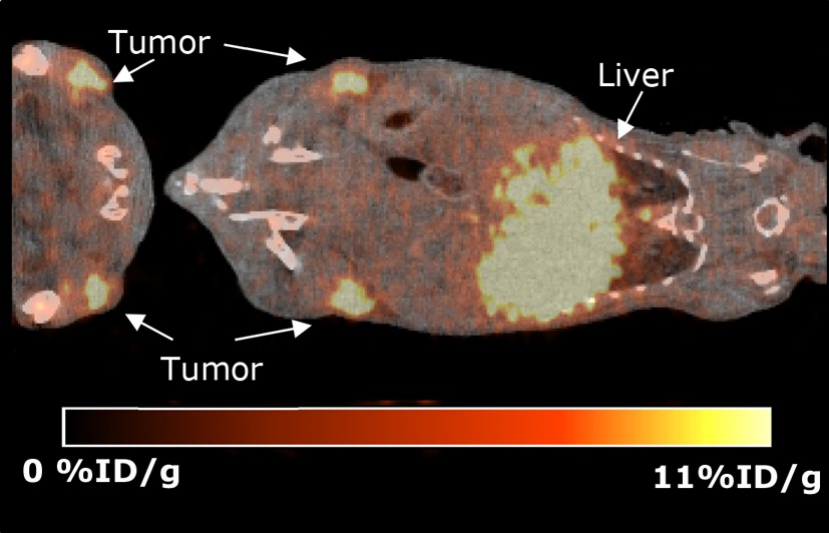
In vivo, AM, PET scan of a dual-flank U-87 MG tumor-bearing mouse 5 days after injection of 1.9 MBq of ^134^Ce-cDTPA-ATN291, 900 s acquisition time.

**Figure 3:**
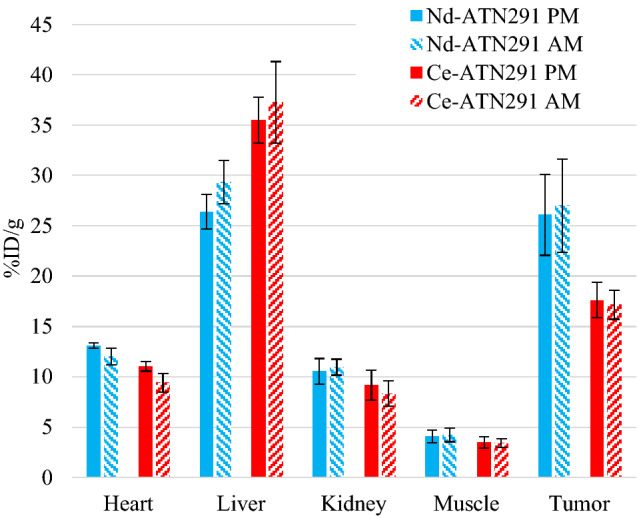
Quantifications for the 5-day AM (daughter) and PM (parent) PET scans for ^134^Ce-cDTPA-ATN-291, and ^140^Nd-cDTPA-ATN-291 in U87 MG tumor bearing mice. The “heart” ROI in the PET scans is representative of the blood pool. N = 5 in all cases, and the error bars represent the inter-subject standard deviation.

**Figure 4:**
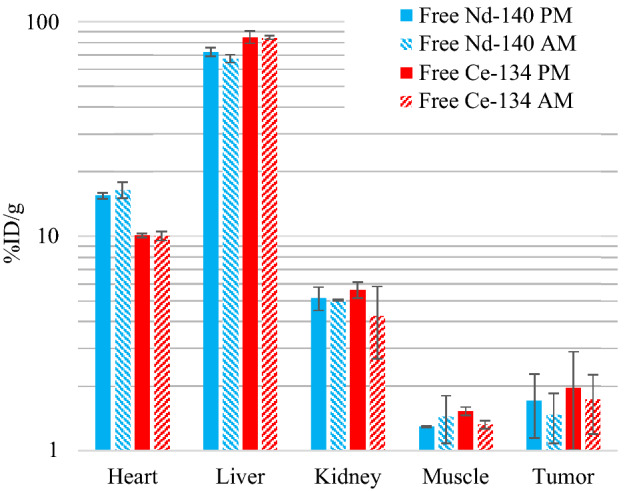
Quantifications for the 4-d AM (daughter) and PM (parent) PET scans for the “free” radionuclides: ^134^Ce and ^140^Nd chlorides formulated in HEPES buffered saline, in U87 MG tumor bearing mice. Data are plotted on a log scale. Note: The “heart” ROI in PET is representative of the blood pool N = 2 in all cases, and the error bars represent the inter-subject standard deviation.

A representative image of ^134^Ce-cDTPA-ATN-291 at 5-days post-injection, *ante-mortem* (AM), is shown in Fig. 2. PET quantifications are given in Figs. 3 and 4 for both the 5-day AM, and *post-mortem* (PM) scans along with the biodistribution measurements for the same tissues. It is important to note that signals from the “heart” ROI include the blood pool in the PET images but only the myocardium was used in the ex vivo biodistribution.

Due to the disruption of chelates during EC decay, the AM images are denoted as tracking the “daughter” while PM images are denoted as tracking the “parent” distributions. In all cases, the daughter is the positron source, however it could be anticipated that in vivo biological processes could move the daughter to a new location before emission of the positron. The 2-h delay between the AM and PM scans allowed any parent-daughter separation that was established in vivo to be re-equilibrated, thereby revealing the location of the tracer-bound parent in the PM scan. In the ^134^Ce/^134^La tomographs from the labeled mAb injections, only the heart (blood pool) ROI was significantly different (2-tailed t-test with p < 0.05) in daughter and parent PET scans (*p* = *0.01, 8 degrees of freedom (d.o.f.)*). For ^140^Nd/^140^Pr imaging from the labeled mAb injections, two regions were significantly different between the AM and PM scans: heart (*p* = *0.02, 8 d.o.f.*) and liver (*p* = *0.04, 8 d.o.f.*).

In each case, the heart signal was significantly lower in the AM scan than in the PM scan. This indicates that ^134^Ce-cDTPA-ATN-291 in circulation, decays and releases ^134^La which is cleared from the blood more rapidly than the intact tracer (and similar for ^140^Nd/^140^Pr). In the ^140^Nd-cDTPA-ATN-291 scans the liver signal was higher in the AM scan than in the PM scan which points towards liver accumulation as the clearance pathway for the free daughter from the blood. This is supported by the biodistributions shown in Fig. 4 from the injections of free ^140^Nd and ^134^Ce in HEPES buffered saline, where accumulation in the liver was the predominant feature. These results are consistent with the findings of Li et al. where the lanthanide DTPA-based complexes exhibited lengthy serum stability and urinary excretion, while less-stable complexes (e.g. La-EDTA) resulted in lanthanide accumulation in liver and bone^16^. The biodistribution in Fig. 3 shows that the Nd and Ce labeled mAbs accumulated differently. This is likely due to lower serum stability for the Ce-cDTPA moiety than for Nd-cDTPA, which is consistent with decreasing Ln-DTPA serum stability with increasing Ln size^16^. Note: significance is not ascribed to the data from free ^134^Ce or ^140^Nd in HEPES buffered saline studies due to the small sample size.

**Figure 5:**
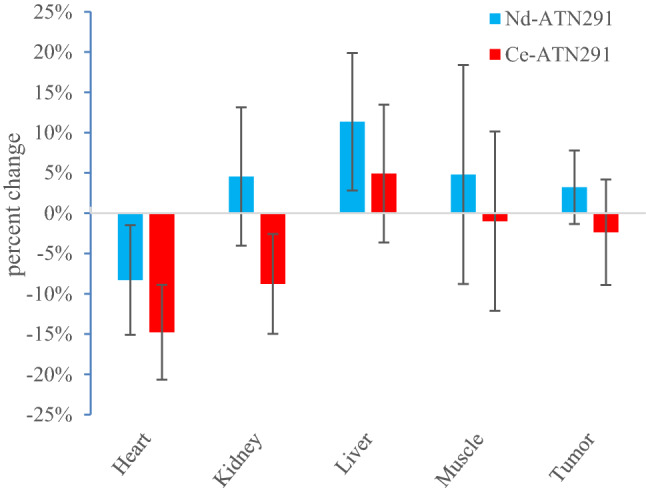
Changes in PET signals due to the parent-daughter separation following EC decay of the parent expressed as percentage differences between daughter (AM) and parent (PM) relative to the parent PET quantification. A negative value indicates that the daughter radioisotope (^140^Pr, or ^134^La) was actively being removed from the ROI while the animal was alive. Error bars represent the standard deviation of the ratio between the daughter and parent signals expressed in percentage. These comparisons are made for the labeled-mAb injections only.

As an alternative comparison, for each animal the ratio of the daughter quantification to the parent quantification in each ROI was computed, and those ratios were averaged over each group. The relative changes between the AM and PM scans are given in Fig. 5, where the ratios are expressed as percentage differences (that is, *[daughter/parent – 1]* × *100%*), and the error bars represent the standard deviation of the ratios. Using this quantification method, an average of ratios, significance against the null hypothesis (that there was no difference between the AM and PM results) is found at the 95% confidence level for the heart (*p* = 0.05) and liver (*p* = 0.04) with the ^140^Nd-cDTPA-ATN-291 tracer, and for the heart (*p* = 0.005) and kidney (*p* = 0.03) when ^134^Ce-cDTPA-ATN-291 was used.

**Figure 6:**
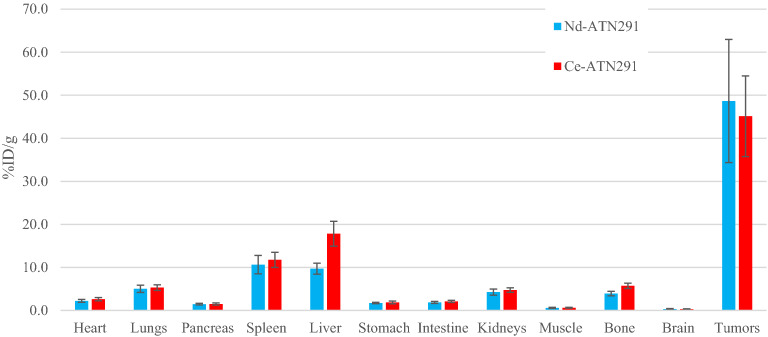
Ex-vivo biodistribution at 5-days after injection with ^140^Nd-cDTPA-ATN-291 or ^134^Ce-cDTPA-ATN-291.

It is important to note that there are stark differences between the excised tissue-based biodistribution shown in Fig. 6 and the PET quantifications shown in Fig. 3. The fact that some tracer remained in circulation at the time of dissection, accounts at least partially for the differences seen in the “heart” (blood pool) ROI PET data compared to the “heart” (myocardium) activity levels in the biodistribution. The remaining difference, notably in the tumor volume, can be attributed to the partial volume effect which was not corrected for in this case. We previously showed a calculation of the partial volume effect for ^140^Nd/^140^Pr, and a similar result can be expected with ^134^Ce/^134^La due to the 1.22 MeV average positron energy in ^134^La decay. The effect was exacerbated in this study due to the small tumor volumes. In future work, a thorough handling of the partial volume correction should be employed in order to allow accurate tissue-based dosimetry for scanning and eventual targeted radionuclide therapy.

### General discussion

Qualitatively, the ATN-291 PET data showed uptake in tissues in U87 MG xenograft bearing mice consistent with the results of Yang et al. for ^89^Zr labeled ATN-291^11^. The tracer distributed primarily to the liver, blood pool, and tumors. Using CT-based regions-of-interest gave smaller relative uptake in tumor tissues than Yang et al. observed, and this is at least partially due to the partial-volume effect discussed above.

When considering ^134^Ce and ^140^Nd as theranostic matched pairs for other hard, 3+ radiometals, the data support that when internalizing vectors are used, the parent-daughter disequilibrium has a minimal effect on qualitative mapping. However, when it comes to performing image-based dosimetry, it is important to recognize that for some tissues the parent tracer distribution is significantly different (p < 0.05) than an in vivo PET image reveals. Additionally, it is critical that any image-based dose mapping be performed after a careful partial volume correction.

Beyond theranostic imaging at late timepoints, these tracers also provide some information about what happens to radiolanthanides when they are released from a targeting vector post-injection. From the data presented here, it is reasonable to conclude that when a radiolanthanide is released from its construct while in the blood stream, a portion of it will redistribute, and its re-distribution will resemble that of the freely injected radiometal in saline. However, with an internalizing vector, radiometals released after accumulation at the tumor site will remain in place. This is consistent with the results presented by McDevitt et al. and bodes well for the prospect of performing targeted radiotherapy with internalizing vectors like ATN-291^2^.

For this work, considerable resources were employed to obtain pure samples of ^134^Ce and ^140^Nd for radiolabeling from CERN-ISOLDE. Since the completion of our study, ISOLDE has completed development of a new capacity for medical isotope production called MEDICIS, which aims to provide additional access to therapeutic and diagnostic radiolanthanides like ^134^La and ^140^Nd (amongst many others) in the background of ISOLDE operation^17^. The MEDICIS methodology and technology is now the basis for an effort across the European Consortium called PRISMAP (*The European Medical Isotope Programme: Production of High Purity Isotopes by Mass Separation for Medical Application*) which should further increase the production capacity for these rare isotopes. In the United States, Bailey et al*.* demonstrated a successful alternative route to ^134^Ce by proton irradiation of ^nat^La metal, resulting in 100 GBq-scale production without the need for mass separation^4^. Based upon these new and ramping production efforts, it is reasonable to anticipate increased availability of ^140^Nd and ^134^Ce in the near future.

## Conclusion

The results presented here support further investigation into employing ^134^Ce and ^140^Nd as diagnostic analogs to therapeutic radiometals like ^177^Lu, ^161^Tb and ^225^Ac. This is especially important because they allow late-timepoint imaging as was performed here at 120 h post injection. However, caution should be taken when performing image-based dosimetry as there is evidence that the daughters ^134^La and ^140^Pr redistribute slightly before emitting their diagnostic positrons. This effect was only significant in a few tissues at the 95% confidence level but should nevertheless be accounted for in dosimetric calculations. The results also support the use of ATN-291 as a carrier mAb for delivering short-ranged therapeutic radionuclides due to its internalizing ability and the subsequent retention of any unbound radiometals in the tumor site.

## Supplementary Information

Supplementary Information.
