# Supplementary material for: PET in vivo generators 134Ce and 140Nd on an internalizing monoclonal antibody probe
Source: Sci Rep. 2022 Mar 9;12:3863. doi: 10.1038/s41598-022-07147-x (PMC8907281; doi:10.1038/s41598-022-07147-x)
Supplement: Supplementary file 1 — Supplementary Information. [file 41598_2022_7147_MOESM1_ESM.pdf]

ARRIVE compliance statement

Organized according to the ARRIVE 2.0 documentation obtained at https://arriveguidelines.org/arrive-guidelines

1. Study Design
    a. For each mouse in the study, the PET scan before sacrifice was compared to the PET scan after sacrifice of the same mouse. Five mice were injected with ^{134}Ce-ATN-291 and five were injected with ^{140}Nd-ATN-291. Additionally, two mice were injected with the unchelated ^{134}Ce and two with the unchelated ^{140}Nd to act as untargeted controls.
    b. The experimental units were single animals, and the results were averaged over the groups (n=5 or n=2 per above).
2. Sample Size
    a. Fourteen mice, total, were used in this work. Five were injected with ^{134}Ce-ATN-291, Five with ^{140}Nd-ATN-291, two with unchelated ^{134}Ce, and two with unchelated ^{140}Nd.
    b. Sample sizes were determined by the amount of anticipated radioactivity available from the collections at CERN-ISOLDE.
3. Exclusion Criteria
    a. For the purposes of imaging animals were to be excluded if tumors were not palpable in both the left and right flanks.
    b. No animals were excluded from the study.
    c. The sample sizes remained consistent with the experimental plan, n=5 for each of the labeled antibody injections and n=2 for each of the non-chelated radiometal injections.
4. Randomization
    a. Animals were randomly assigned for injection, and no preselection was performed for each group.
    b. Confounders were not anticipated, and therefore confounders were not controlled.
5. Blinding
    a. PET ROIs were drawn using the CT images for tissue delineation without visualizing the PET signals. No other blinding was used in the study or analysis.
6. Outcome Measures
    a. The measured dependent variables were the tissue-based %ID/g in the PET and biodistribution studies. The “intervention” was the sacrifice of each animal, effectively ceasing the parent-daughter disequilibrium induced by biological processes. The primary outcome measure was the ratio of the pre-intervention to post intervention relative PET signals, and ultimately the level of statistical significance between that ratio and the null hypothesis (that the ratio was equal to 1.0) was the reported result.
    b. The primary hypothesis being tested was that due to the internalization of the ATN-291 antibody, a parent-daughter disequilibrium would not be established in the tumor tissue. Due to the small sample size (determined by the amount of available radiotracer), the study provided *p*-values to indicate the level of statistical significance of the data rather than conclusively affirming or disaffirming the hypothesis.
7. Statistical Methods
    a. Two-tailed t-tests were performed to determine the p-values reported. For the purposes of data interpretation, p values less than 0.05 (95% C.I.) were denoted as

significant, but the p-values were provided to allow the reader to make their own interpretation.

   b. The appropriateness of the statistical approach was not tested.

8. Experimental Animals
   a. All animals in this study were female NMRI nude mice (Taconic, Denmark).
   b. Besides the inoculation with tumor cells as described in the text, no other relevant procedures were performed on the animals.
9. Experimental procedures: The experimental procedures are described in the text, for additional information contact Greg Severin, gwseverin@chemistry.msu.edu
10. Results: The results and statistical interpretation are provided in the text, for additional information contact Greg Severin, gwseverin@chemistry.msu.edu
